# The Hospital Burden Associated With Intergenerational Contact With the Welfare System in Australia

**DOI:** 10.1001/jamanetworkopen.2022.26203

**Published:** 2022-08-05

**Authors:** Alexandra M. Procter, Catherine R. Chittleborough, Rhiannon M. Pilkington, Odette Pearson, Alicia Montgomerie, John W. Lynch

**Affiliations:** 1 School of Public Health, The University of Adelaide, Adelaide, Australia; 2 Robinson Research Institute, The University of Adelaide, Adelaide, Australia; 3 Wardliparingga Aboriginal Health Research Unit, South Australian Health and Medical Research Institute, Adelaide, Australia; 4 Adelaide Medical School, The University of Adelaide, Adelaide, Australia; 5 Population Health Sciences, University of Bristol, Bristol, United Kingdom

## Abstract

**Importance:**

Intergenerational welfare contact is a policy issue because of the personal and social costs of entrenched disadvantage; yet, few studies have quantified the burden associated with intergenerational welfare contact for health.

**Objective:**

To examine the proportion of individuals who experienced intergenerational welfare contact and other welfare contact types and to estimate their cause-specific hospital burden.

**Design, Setting, and Participants:**

This cohort study used a whole-of-population linked administrative dataset of individuals followed from birth to age 20 years using deidentified data from the Better Evidence Better Outcomes Linked Data platform (Australian Government Centrelink [welfare payments], birth registration, perinatal birth records, and inpatient hospitalizations). Participants included individuals born in South Australia from 1991 to 1995 and their parents. Analysis was undertaken from January 2020 to June 2022.

**Exposures:**

Using Australian Government Centrelink data, welfare contact was defined as 1 or more parents receiving a means-tested welfare payment (low-income, unemployment, disability, or caring) when children were aged 11 to 15 years, or youth receiving payment at ages 16 to 20 years. Intergenerational welfare contact was defined as welfare contact occurring in both parent and offspring generations. Offspring were classified as: no welfare contact, parent-only welfare contact, offspring-only welfare contact, or intergenerational welfare contact.

**Main Outcomes and Measures:**

Hospitalization rates and cumulative incidence were estimated by age, hospitalization cause, and welfare contact group.

**Results:**

A total of 94 358 offspring (48 589 [51.5%] male) and 143 814 parents were included in analyses. The study population included 32 969 offspring (34.9%) who experienced intergenerational welfare contact. These individuals were more socioeconomically disadvantaged at birth and had the highest hospitalization rate (133.5 hospitalizations per 1000 person-years) compared with individuals with no welfare contact (46.1 hospitalizations per 1000 person-years), individuals with parent-only welfare contact (75.0 hospitalizations per 1000 person-years), and individuals with offspring-only welfare contact (87.6 hospitalizations per 1000 person-years). Hospitalizations were frequently related to injury, mental health, and pregnancy. For example, the proportion of individuals with intergenerational welfare contact who had experienced at least 1 hospitalization at ages 16 to 20 years was highest for injury (9.0% [95% CI, 8.7%-9.3%]).

**Conclusions and Relevance:**

In this population-based cohort study, individuals who experienced intergenerational welfare contact represented one-third of the population aged 11 to 20 years. Compared with individuals with parent-only welfare contact, individuals with intergenerational welfare contact were more disadvantaged at birth and had 78% higher hospitalization rates from age 11 to 20 years, accounting for more than half of all hospitalizations. Frequent hospitalization causes were injuries, mental health, and pregnancy. This study provides the policy-relevant estimate for what it could mean to break cycles of disadvantage for reducing hospital burden.

## Key Points

**Question:**

What is the hospital burden associated with individuals who experience intergenerational contact with the welfare system?

**Findings:**

In this cohort study of 94 358 individuals born in South Australia from 1991 to 1995, and 143 814 parents, approximately one-third (34.9%) of the population experienced intergenerational welfare contact. Individuals who experienced intergenerational welfare contact had a higher hospitalization rate (133.5 hospitalizations per 1000 person-years) compared with individuals whose parents experienced welfare contact but they did not (75.0 hospitalizations per 1000 person-years).

**Meaning:**

This cohort study provides the policy relevant estimate for what it could mean to break cycles of disadvantage for reducing hospital burden.

## Teaser

This cohort study examines the proportion of individuals who experienced intergenerational welfare contact and other welfare contact types and estimates their cause-specific hospital burden.

## Introduction

In 1848, Friedrich Engels^1^ questioned “How is it possible, under such conditions, for the lower class to be healthy and long lived?” Even with major population health advances since the 19th century, health gaps between rich and poor remain entrenched.^2^ In 2017 to 2018, 1 in 6 children in Australia aged younger than 15 years were living in poverty, defined as less than half the population median household income.^3^ Growing up in socioeconomic disadvantage negatively affects child development, education, labor market, and health outcomes over a life course and across generations.^4,5,6^ Income provides access to resources, such as nutrition, housing, and medical care, which directly and indirectly affect well-being and is a major social determinant of health.^4,7,8^

In high- and middle-income countries, welfare systems have the potential to buffer the effects of low income. Welfare systems, including Centrelink in Australia, aim to transfer income to support those experiencing low income due to joblessness, disability, retirement, and caring responsibilities.^9,10^ Payments are intended to help overcome the income loss from these circumstances.^9,11^ More than 40% of Australia’s cash transfers are targeted at the lowest income quintile, making it the second highest targeted cash transfer system of all Organization for Economic Cooperation and Development countries. Such a highly targeted welfare system supports the notion that welfare receipt is closely related to ongoing poverty and social exclusion.^12^

There is concern in Australia^13,14,15^ and internationally^16,17,18^ regarding families who become “trapped” in a cycle of intergenerational welfare contact (IWC). Policy discussion for government and nongovernment organizations has been about breaking these cycles of disadvantage.^13,19,20^ We used this conceptualization of “breaking the cycle” of IWC to guide our analysis. We selected groups of welfare contact (WC) to conceptually mirror how they have been framed in policy discussions and identify our main groups of interest: individuals who experience parent-only WC and individuals who go on to experience IWC. This reflects that any intervention to prevent IWC would rely on targeting families with parent WC as the population at risk of progressing into IWC. The idea that reliance on welfare is transmitted from one generation to the next is not unique to Australia^11,18,21^ and has been documented in the United States,^22,23^ Canada,^22^ Nordic countries,^24,25^ and United Kingdom.^26^ Despite considerable research documenting that low income is associated with poor health,^4,7,8,27^ few studies examine IWC and health.^11,28^ One study investigated the mediating role of health (limited to body mass index and self-reported mental health and physical exercise) in IWC but did not report health outcomes for individuals with IWC.^28^ Previous studies reporting that WC is associated with poorer health are limited by quality (eg, self-reported measures,^29,30^ selective sample populations,^28,29,30,31^ no longitudinal design^15^), lack of intergenerational perspective,^29,31,32,33,34,35^ and WC definitions that include any payment type, such as those considered near-universal.^11,30,31,32,33,34,35^

The policy problem of IWC in Australia has been examined by labor economist Cobb-Clark and colleagues,^21^ who defined and measured IWC to better understand how disadvantage is transmitted from parents to offspring. Using welfare payment eligibility criteria, we build on this to develop our preferred definition of means-tested WC that includes welfare payments that are intended to support individuals maintaining a basic standard of living.

The objective of our study is descriptive.^36^ We use whole-of-population linked administrative data that contains Australian government welfare payments linked to jurisdictional inpatient hospitalizations. To our knowledge, this is the first time welfare payment data has been linked to state hospital data in Australia. This linked data cohort study examined the proportion of almost 100 000 individuals in South Australia born from 1991 to 1995 who experienced IWC and other WC types; and their cause-specific hospitalization experience from ages 11 to 20 years. We measured WC for parents when their offspring were aged 11 to 15 years, and for the offspring themselves receiving payment at ages 16 to 20 years. These birth cohorts and age ranges were selected to maximize the exposure (WC) and outcome (hospitalization) windows, given the data structure involving multiple birth cohorts (eFigure 1 in the Supplement). While the data platform used is whole-of-population, we acknowledge that this research has not included governance by Aboriginal and Torres Strait Islander people; therefore, we do not include data on Indigenous individuals and families.^37^

## Methods

For this cohort study, ethics approval for use of data was granted from the University of Adelaide, the South Australian Department for Health and Wellbeing, and the Australian Institute of Health and Welfare (AIHW) Ethics Committee. Site-specific approval for use of hospital data was given by South Australian Department for Health and Wellbeing and Women’s and Children’s Health Network. A waiver of consent was granted pursuant to section S95 of the Australian Government’s Privacy Act 1988 by the AIHW Ethics Committee and under the National Statement on Ethical Conduct in Human Research by the South Australian Department for Health and Wellbeing. This study is reported following the Strengthening the Reporting of Observational Studies in Epidemiology (STROBE) reporting guideline.

### Data Sources

Data were part of the Better Evidence Better Outcomes through Linked Data (BEBOLD) platform, which is a whole-of-population administrative data resource.^38^ We used inpatient hospitalization (Integrated South Australian Activity Collection); South Australian Perinatal Statistics Collection; South Australian Births, Death and Marriages Registry; family file (linking parents to children through birth registrations); and Centrelink data. Probabilistic linkage was performed by SA-NT DataLink, an independent third-party linkage agency.^39^ Australian data linkage systems estimate false linkage rates from 0.1%^40^ to 0.5%.^41^ Centrelink data (Data Over Multiple Individual Occurrences [DOMINO]) were linked to BEBOLD by the AIHW. DOMINO contains deidentified individual event-based data to provide a longitudinal picture of individual welfare payments by the Commonwealth Department of Social Services.

### Population

Individuals in South Australia born from 1991 to 1995 were eligible to be included in the study (eFigure 2 in the Supplement). Individuals were identified as Aboriginal and/or Torres Strait Islander if they had at least 1 record as Aboriginal and/or Torres Strait Islander across inpatient hospitalization, South Australian Perinatal Statistics, Centrelink demographics (DOMINO), and South Australian Births Registry.^42^ We made a conscious decision, in consultation with our Aboriginal and Torres Strait Islander coauthor (O.P.) and informed by the principles of Indigenous Data Sovereignty, to not include Aboriginal and Torres Strait Islander individuals.^37^ We also excluded individuals who did not have a birth registration with at least 1 parent recorded and parents who were unable to be linked owing to missing information. Analysis was undertaken on individuals who were neither Aboriginal nor Torres Strait Islander and their parents. We defined individuals’ parents using birth registration data, where mother and (if acknowledged) coparent was recorded.

### Indicators of Socioeconomic Disadvantage at Birth

Indicators of socioeconomic disadvantage at birth were sourced from the South Australian Perinatal Statistics Collection,^43^ supplemented by South Australian Birth Registry data. Characteristics examined are listed in eTable 1 in the Supplement.

### Hospitalizations

Inpatient hospitalization data record age at admission and the *International Statistical Classification of Diseases and Related Health Problems, Tenth Revision, Australian Modification* (*ICD-10-AM*) codes for the admission.^44^ Hospitalization type by chapter heading of the *ICD-10-AM* was defined using the primary diagnosis code recorded for the admission.^44^ Potentially preventable hospitalizations (PPHs) were calculated under a child-centric definition for admission at ages 11 to 14 years and under the standard Australian definition (best suited to adults) at ages 15 to 20 years.^45^ Primary diagnosis codes used to identify pediatric Complex Chronic Conditions (CCCs) were adapted to the *ICD-10-AM* coding.^46^

### Welfare Contact

WC was defined as having received a Centrelink payment in which the payment amount was greater than $0 and the payment type was recorded (0.003% had a missing payment type). Parent WC was defined as parents having WC when children were aged 11 to 15 years, and offspring WC as the offspring themselves having WC when aged 16 to 20 years (eFigure 1 in the Supplement). IWC was WC occurring in both parent and offspring generations. Individuals were categorized in 1 of 4 WC groups: no WC, parent-only WC, offspring-only WC, and IWC.

We examined WC under 2 constructs: means-tested WC included only payments subject to an income and assets test aimed at assisting those experiencing financial hardship, and any WC included all payment types, including those considered near universal for families with children (eg, Family Tax Benefit). Included payment types under our definition of means-tested WC and the intent of these payments are detailed in eTable 2 in the Supplement.

### Statistical Analysis

For each birth cohort, we calculated the proportion of individuals who experienced no WC, parent-only WC, offspring-only WC, and IWC, under both welfare constructs (any WC and means-tested WC). We calculated the proportion of offspring with indicators of socioeconomic disadvantage at birth by WC type.

All-cause age-specific and overall admission rates for individuals aged 11 to 25 years were calculated by dividing the number of admissions by person-years. Cumulative incidence was the proportion of individuals who had experienced a hospital admission from age 11 to 20 years. Hospitalization admission rates and proportion of individuals experiencing hospitalization by *ICD-10-AM* chapter, PPHs, and CCCs were calculated for ages 11 to 15 years, when offspring could have been exposed to parent WC, and 16 to 20 years, reflecting the age group when individuals could have experienced their own WC. Hospitalization outcomes were reported across WC types under the means-tested welfare construct.

Given the data structure and years of observation available, we undertook sensitivity analysis to assess potential misclassification due to the limited observation windows for both parent and offspring WC under the means-tested welfare construct. For individuals born in 1991, we examined offspring WC for an extended period (ages 16-24 years) and compared this with offspring WC at ages 16 to 20 years. For individuals born in 1995, we examined parent WC for an extended period (ages 7-15 years) and compared this with parent WC at ages 11 to 15 years. For both birth cohorts, we recreated the 4 WC groups using the extended period of parent or offspring WC.

Given that family structures can change over time, we calculated the proportion of individuals whose parents were defined by birth registration (recorded at the time of birth) compared with parents recorded in the Centrelink relationships file (recorded at time of payment receipt). We defined *parents* in the Centrelink relationships file as parent, grandparent, or guardian relationships associated with the individual. Gender of Centrelink parent relationship was defined using the gender recorded in the Centrelink demographics file. This sensitivity analysis was undertaken for individuals born between 1995 and 2002.

Analyses were conducted using Stata MP statistical software version 16, 64-bit (StataCorp). Analysis was undertaken in January 2020 to June 2022.

## Results

A total of 98 571 individuals born in South Australia from 1991 to 1995 were eligible for inclusion. Of these, we excluded 4103 Aboriginal and/or Torres Strait Islander individuals and 110 individuals whose birth registration did not record at least 1 parent. The final cohort included 94 358 offspring (48 589 [51.5%] male) and 143 814 parents. Of offspring analyzed, 91 192 (96.6%) had both a mother and coparent recorded in birth registration data and 3166 (3.4%) had 1 parent recorded.

### Welfare Contact

Using any type of welfare payment, nearly half of included offspring (46 147 offspring [48.9%]) experienced IWC, and only 9269 offspring (9.8%) experienced no WC (Table 1). Using the means-tested definition, IWC was experienced by one-third of offspring (32 969 offspring [34.9%]), with 37 507 offspring (39.7%) experiencing no WC, 10 260 offspring (10.9%) experiencing parent-only WC, and 13 622 offspring (14.4%) offspring-only WC. Offspring experiencing IWC were more socioeconomically disadvantaged at birth compared with any other WC group (eTable 1 in the Supplement).

**Table 1.** Welfare Contact for Individuals Born 1991 to 1995

Year of birth	Welfare contact group, No. (%) [95% CI] (N = 94 358)				Overall, No.
	None	Parent only	Offspring only	Intergenerational	
**Any welfare payment**					
1991	1988 (10.6) [10.2-11.0]	7438 (39.7) [39.0-40.4]	437 (2.3) [2.1-2.5]	8881 (47.4) [46.7-48.1]	18 744
1992	1911 (10.0) [9.6-10.4]	7536 (39.4) [38.7-40.1]	339 (1.8) [1.6-2.0]	9335 (48.8) [48.1-49.5]	19 121
1993	1772 (9.3) [8.9-9.7]	7558 (39.8) [39.1-40.5]	294 (1.5) [1.3-1.7]	9388 (49.4) [48.7-50.1]	19 012
1994	1815 (9.6) [9.2-10.0]	7226 (38.4) [37.7-39.1]	268 (1.4) [1.2-1.6]	9525 (50.6) [49.9-51.3]	18 834
1995	1783 (9.6) [9.2-10.0]	7584 (40.7) [40.0-41.4]	262 (1.4) [1.2-1.6]	9018 (48.4) [47.7-49.1]	18 647
Overall	9269 (9.8) [9.6-10.0]]	37 342 (39.6) [39.3-39.9]	1600 (1.7) [1.6-1.8]	46 147 (48.9) [48.6-49.2]	94 358
**Means-tested welfare payment** ^a^					
1991	7470 (39.9) [39.2-40.6	2231 (11.9) [11.4-12.4]	2517 (13.4) [12.9-13.9]	6526 (34.8) [34.1-35.5]	18 744
1992	7554 (39.5) [38.8-40.2]	2193 (11.5) [11.0-12.0]	2673 (14.0) [13.5-14.5]	6701 (35.0) [34.3-35.7]	19 121
1993	7477 (39.3) [38.6-40.0]	2078 (10.9) [10.5-11.3]	2858 (15.0) [14.5-15.5]	6599 (34.7) [34.0-35.4]	19 012
1994	7397 (39.3) [38.6-40.0]	1845 (9.8) [9.4-10.2]	2835 (15.1) [14.6-15.6]	6757 (35.9) [35.2-36.6]	18 834
1995	7609 (40.8) [40.1-41.5]	1913 (10.3) [9.9-10.7]	2739 (14.7) [14.2-15.2]	6386 (34.2) [33.5-34.9]	18 647
Overall	37 507 (39.7) [39.4-40.0]	10 260 (10.9) [10.7-11.1]	13 622 (14.4) [14.2-14.6]	32 969 (34.9) [34.6-35.2]	94 358

^a^ Means-tested contains the following payment types: Carer Allowance, Carer Payment, Disability Support Pension, Newstart Allowance, Newstart Mature Age Allowance, Parenting payment partnered, Parenting payment single, Partner allowance, Wife Pension Disability Support Pension (wife of a Disability Support pension), and Youth Allowance.

### All-Cause Hospitalizations

From the perspective of health system burden as incidents of hospitalization, the all-cause hospitalization rate among individuals aged 11 to 25 years was 46.1 hospitalizations per 1000 person-years among those with no WC, 75.0 hospitalizations per 1000 person-years among those with parent-only welfare contact, 87.6 hospitalizations per 1000 person-years among those with offspring-only welfare contact, and 133.5 hospitalizations per 1000 person-years for individuals with IWC (Table 2). For every age, individuals with IWC had higher admission rates compared with individuals experiencing no WC, or WC in only 1 generation, with the exception of age 11, when individuals with IWC and those with parent-only WC had similar hospitalization rates. From the perspective of a cohort of individuals experiencing hospitalization, the cumulative incidence (proportion of individuals hospitalized at least once) from ages 11 to 20 years was highest for individuals with IWC (43.0% [95% CI, 42.4%-43.6%]) compared with individuals experiencing no WC (23.7% [95% CI, 23.2%-24.2%]), parent-only WC (29.3% [95% CI, 28.8%-29.8%]), or offspring-only WC (34.3% [95% CI, 33.8%-34.8%]) (Table 3).

**Table 2.** All-Cause Hospital Admission Rates for Individuals Born 1991 to 1995 by Welfare Contact^a^

Birth cohort	Welfare contact group									
	None		Parent only		Offspring only		Intergenerational		Overall	
	No.	Rate per 1000 PY (95% CI)	No.	Rate per 1000 PY (95% CI)	No.	Rate per 1000 PY (95% CI)	No.	Rate per 1000 PY (95% CI)	No.	Rate per 1000 PY (95% CI)
1991-1995, age, y										
11	1066	28.4 (26.7-30.1)	692	67.4 (62.5-72.3)	510	37.4 (34.2-40.6)	2161	65.5 (62.8-68.2)	4429	46.9 (45.6-48.2)
12	1187	31.6 (29.8-33.4)	615	59.9 (55.3-64.5	613	45.0 (41.5-48.5)	2184	66.2 (63.5-68.9)	4599	48.7 (47.3-50.1)
13	1275	34.0 (32.2-35.8)	664	64.7 (59.9-69.5	624	45.8 (42.3-49.3)	2335	70.8 (68.0-73.6)	4898	51.9 (50.5-53.3)
14	1377	36.7 (34.8-38.6)	766	74.7 (69.6-79.8)	687	50.4 (46.7-54.1)	2845	86.3 (83.3-89.3)	5675	60.1 (58.6-61.6)
15	1496	39.9 (37.9-41.9)	766	74.7 (69.6-79.8)	855	62.8 (58.7-66.9)	3401	103.2 (99.9-106.5	6518	69.1 (67.5-70.7)
16	1669	44.5 (42.4-46.6)	732	71.3 (66.3-76.3)	1275	93.6 (88.7-98.5)	4275	129.7 (126.1-133.3)	7951	84.3 (82.5-86.1)
17	1892	50.4 (48.2-52.6))	874	85.2 (79.8-90.6)	1339	98.3 (93.3-103.3)	4842	146.9 (143.1-150.7)	8947	94.8 (92.9-96.7)
18	1969	52.5 (50.2-54.8	707	68.9 (64.0-73.8)	1463	107.4 (102.2-112.6)	5242	159.0 (155.1-162.9)	9381	99.4 (97.5-101.3)
19	1954	52.1 (49.9-54.3	724	70.6 (65.6-75.6)	1546	113.5 (108.2-118.8)	5637	171.0 (166.9-175.1)	9861	104.5 (102.5-106.5)
20	1974	52.6 (50.3-54.9	771	75.1 (70.0-80.2)	1667	122.4 (116.9-127.9)	5691	172.6 (168.5-176.7)	10 103	107.1 (105.1-109.1)
21	2130	56.8 (54.5-59.1)	851	82.9 (77.6-88.2)	1747	128.2 (122.6-133.8)	5568	168.9 (164.9-172.9)	10 296	109.1 (107.1-111.1)
1991-1994 (age 22 y)	1650	55.2 (52.6-57.8)	730	87.5 (81.4-93.6)	1268	116.5 (110.5-122.5)	4917	185.0 (180.3-189.7)	8565	113.1 (110.8-115.4)
1991-1993 (age 23 y	1372	61.0 (57.9-64.1)	643	98.9 (91.6-106.2)	952	118.3 (111.2-125.4)	3903	196.9 (191.4-202.4)	6870	120.8 (118.1-123.5)
1991-1992 (age 24 y	916	61.0 (57.2-64.8)	360	81.4 (73.3-89.5)	621	119.7 (110.9-128.5)	2774	209.7 (202.8-216.6)	4671	123.4 (120.1-126.7)
1991 (age 25 y)	523	70.0 (64.2-75.8)	189	84.7 (73.1-96.3)	286	113.6 (101.2-126.0)	1488	228.0 (217.8-238.2)	2486	132.6 (127.7-137.5)
Total	22 450	46.1 (45.5-46.7)	10 084	75.0 (73.6-76.4)	15 453	87.6 (86.3-88.9)	57 263	133.5 (132.5-134.5)	105 250	85.8 (85.3-86.3)

Abbreviation: PY, person-years.

^a^ Analyses included 94 358 individuals, 105 250 hospitalizations, and 1 227 135 person-years. Person-years for each age and WC type is presented in eTable 7 in the Supplement.

**Table 3.** Cumulative Incidence of All-Cause Hospitalization by Welfare Contact and Age

Age, y	Welfare contact group, No. (%) [95% CI]				
	None (n = 37 507)	Parent only- (n = 10 260)	Offspring only (n = 13 622)	Intergenerational (n = 32 969)	Overall, No. (%) (N = 94 358)
11	875 (2.3) [1.8-2.8]	367 (3.6) [3.0-4.2]	417 (3.1) [2.5-3.7]	1598 (4.8) [4.1-5.5]	3257 (3.5)
12	1734 (4.6) [4.1-5.1]	671 (6.5) [5.9-7.1]	847 (6.2) [5.6-6.8]	2853 (8.7) [8.0-9.4]	6105 (6.5)
13	2528 (6.7) [6.2-7.2]	978 (9.5) [8.9-10.1]	1216 (8.9) [8.3-9.5]	4069 (12.3) [11.6-13.0]	8791 (9.3)
14	3404 (9.1) [8.6-9.6]	1263 (12.3) [11.7-12.9]	1610 (11.8) [11.2-12.4]	5404 (16.4) [15.7-17.1]	11 681 (12.4)
15	4301 (11.5) [11.0-12.0]	1585 (15.4) [14.8-16.0]	2066 (15.2) [14.6-15.8]	6891 (20.9) [20.2-21.6]	14 843 (15.7)
16	5219 (13.9) [13.4-14.4]	1898 (18.5) [17.9-19.1]	2577 (18.9) [18.3-19.5]	8461 (25.7) [25.1-26.3]	18 155 (19.2)
17	6190 (16.5) [16.0-17.0]	2212 (21.6) [21.1-22.1]	3151 (23.1) [22.5-23.7]	9987 (30.3) [29.7-30.9]	21 540 (22.8)
18	7128 (19.0) [18.5-19.5]	2506 (24.4) [23.9-24.9]	3728 (27.4) [26.8-28.0]	11 526 (35.0) [34.4-35.6]	24 888 (26.4)
19	8003 (21.3) [20.8-21.8]	2773 (27.0) [26.5-27.5]	4248 (31.2) [30.7-31.7]	12 888 (39.1) [38.5-39.7]	27 912 (29.6)
20	8873 (23.7) [23.2-24.2]	3009 (29.3) [28.8-29.8]	4679 (34.3) [33.8-34.8]	14 190 (43.0) [42.4-43.6]	30 751 (32.6)

### Cause-Specific Hospitalizations

The proportion of individuals with at least 1 hospitalization by condition type (*ICD-10-AM* chapter heading, PPHs, and CCCs) and WC group are shown for hospitalizations among individuals ages 11 to 15 years (Table 4) and 16 to 20 years (Table 5). The most common causes of admission were injuries, poisoning, and external causes regardless of WC or age group, but the proportion of individuals who had experienced at least 1 hospitalization related to injury was highest among those with IWC (9.0% [95% CI, 8.7%-9.3%]). A higher proportion of individuals with IWC experienced at least 1 hospitalization related to respiratory conditions, mental health, digestive conditions, and pregnancy compared with any other type of WC for both age groups. For these causes, the proportion was higher at ages 16 to 20 years compared with ages 11 to 15 years (eg, Mental and Behavioral disorders [F00-F99]: age 11-15 years: 1.4% [95% CI, 1.3%-1.5%]; age 16-20 years: 3.7% [95% CI, 3.5%-3.9%]). Individuals with IWC had the highest proportion of PPHs compared with individuals experiencing all other WC types (age 11-15 years: 4.5% [95% CI, 4.3%-4.7%]; age 16-20 years: 4.8% [95% CI, 4.6%-5.0%]). Only a small proportion of individuals with IWC experienced CCCs across both age groups (age 11-15 years: 1.9% [95% CI, 1.8%-2.0%]; age 16-20 years: 2.2% [95% CI, 2.0%-2.4%]). Similar patterns were observed when taking a health system perspective by reporting the cause-specific (*ICD-10-AM* chapter heading, PPHs, and CCCs) admission rates per 1000 person-years by WC and age group (eTable 3 and eTable 4 in the Supplement).

**Table 4.** Proportion of Individuals With at Least 1 Hospitalization by Condition Type and Welfare Contact for Individuals Born 1991 to 1995, Aged 11 to 15 Years^a^

Condition type	*ICD-10-AM* codes	Welfare contact group, No. (%) [95% CI]				
		None (n = 37 507)	Parent only (n = 10 260)	Offspring only (n = 13 622)	Intergenerational (n = 32 969)	Overall (N = 94 358)
Certain infectious and parasitic diseases	A00-B99	283 (0.8) [0.7-0.9]	109 (1.1) [0.9-1.3]	128 (0.9) [0.7-1.0]	425 (1.3) [1.2-1.4]	945 (1.0) [0.9-1.1]
Neoplasms	C00-D48	92 (0.2) [0.2-0.2]	84 (0.8) [0.6-1.0]	65 (0.5) [0.4-0.6]	229 (0.7) [0.6-0.8]	470 (0.5) [0.5-0.5]
Diseases of the blood and blood-forming organs and certain disorders involving the immune mechanism	D50-D89	24 (0.1) [0.1-0.1]	26 (0.3) [0.2-0.4]	9 (0.1) [0.0-0.2]	41 (0.1) [0.1-0.1]	100 (0.1) [0.1-0.1]
Endocrine, nutritional, and metabolic disorders	E00-E89	90 (0.2) [0.2-0.2]	79 (0.8) [0.6-1.0]	41 (0.3) [0.2-0.4]	197 (0.6) [0.5-0.7]	407 (0.4) [0.4-0.4]
Mental and behavioral disorders	F00-F99	151 (0.4) [0.3-0.5]	50 (0.5) [0.4-0.6]	88 (0.6) [0.5-0.7]	457 (1.4) [1.3-1.5]	746 (0.8) [0.7-0.9]
Diseases						
Nervous system	G00-G99	112 (0.3) [0.2-0.4]	51 (0.5) [0.4-0.6]	52 (0.4) [0.3-0.5]	285 (0.9) [0.8-1.0]	500 (0.5) [0.5-0.5]
Eye and adnexa	H00-H59	34 (0.1) [0.1-0.1]	8 (0.1) [0.0-0.2]	17 (0.1) [0.0-0.2]	87 (0.3) [0.2-0.4]	146 (0.2) [0.2-0.2]
Ear and mastoid process	H60-H95	71 (0.2) [0.2-0.2]	41 (0.4) [0.3-0.5]	48 (0.4) [0.3-0.5]	216 (0.7) [0.6-0.8]	376 (0.4) [0.4-0.4]
Circulatory system	I00-I99	71 (0.2) [0.2-0.2]	34 (0.3) [0.2-0.4]	34 (0.2) [0.1-0.3]	120 (0.4) [0.3-0.5]	259 (0.3) [0.3-0.3]
Respiratory system	J00-J99	504 (1.3) [1.2-1.4]	222 (2.2) [1.9-2.5]	318 (2.3) [2.0-2.6]	1171 (3.6) [3.4-3.8]	2215 (2.3) [2.2-2.4]
Digestive system	K00-K93	680 (1.8) [1.7-1.9]	252 (2.5) [2.2-2.8]	365 (2.7) [2.4-3.0]	1112 (3.4) [3.2-3.6]	2409 (2.6) [2.5-2.7]
Skin and subcutaneous tissue system	L00-L099	214 (0.6) [0.5-0.7]	77 (0.8) [0.6-1.0]	128 (0.9) [0.7-1.1]	500 (1.5) [1.4-1.6]	919 (1.0) [0.9-1.1]
Musculoskeletal system and connective tissue	M00-M99	205 (0.5) [0.4-0.6]	73 (0.7) [0.5-0.9]	106 (0.8) [0.7-0.9]	385 (1.2) [1.1-1.3]	769 (0.8) [0.7-0.9]
Genitourinary system	N00-N99	204 (0.5) [0.4-0.6]	84 (0.8) [0.6-1.0]	98 (0.7) [0.6-0.8]	354 (1.1) [1.0-1.2]	740 (0.8) [0.7-0.9]
Pregnancy, childbirth, and the puerperium	O00-O99	7 (<0.1) [<0.1-<0.1]	6 (0.1) [0.0-0.2]	21 (0.2) [0.1-0.3]	145 (0.4) [0.3-0.5]	179 (0.2) [0.2-0.2]
Certain conditions originating in the perinatal period	P00-P96	0	0	0	0	0
Congenital malformations, deformations, and chromosomal abnormalities	Q00-Q99	87 (0.2) [0.2-0.2]	37 (0.4) [0.3-0.5]	39 (0.3) [0.2-0.4]	205 (0.6) [0.5-0.7]	368 (0.4) [0.4-0.4]
Symptoms, signs, and abnormal clinical and laboratory findings, not elsewhere classified	R00-R99	452 (1.2) [1.1-1.3]	161 (1.6) [1.4-1.8]	211 (1.5) [1.3-1.7]	846 (2.6) [2.4-2.8]	1670 (1.8) [1.7-1.9]
Injury, poisoning, and certain other consequences of external causes	S00-T98	1670 (4.5) [4.3-4.7]	554 (5.4) [5.0-5.8]	710 (5.2) [4.8-5.6]	2174 (6.6) [6.3-6.9]	5108 (5.4) [5.3-5.5]
Factors influencing health status and contact with health services	Z00-Z99	208 (0.6) [0.5-0.7]	102 (1.0) [0.8-1.2]	87 (0.6) [0.5-0.7]	328 (1.0) [0.9-1.1]	725 (0.8) [0.7-0.9]
PPHs	NA	726 (1.9) [1.8-2.0]	296 (2.9) [2.6-3.2]	364 (2.7) [2.4-3.0]	1485 (4.5) [4.3-4.7]	2871 (3.0) [2.9-3.1]
CCCs	NA	164 (0.4) [0.3-0.5]	208 (2.0) [1.7-2.3]	79 (0.6) [0.5-0.7]	630 (1.9) [1.8-2.0]	1081 (1.1) [1.0-1.2]

Abbreviations: CCC, complex chronic condition; *ICD-10-AM*, *International Statistical Classification of Diseases and Related Health Problems, Tenth Revision, Australian Modification*; NA, not applicable; PPH; potentially preventable hospitalization.

^a^ Individuals may experience multiple admissions and therefore may fall into multiple *ICD-10-AM* chapters.

**Table 5.** Proportion of Individuals With at Least 1 Hospitalization by Condition Type and Welfare Contact for Individuals Born From 1991 to 1995, Aged 16 to 20 Years^a^

Condition type	*ICD-10-AM* codes	Welfare contact group, No. (%) [95% CI]				
		None (n = 37 507)	Parent only (n = 10 260)	Offspring only (n = 13 622)	Intergenerational (n = 32 969)	Overall (N = 94 358)
Certain infectious & parasitic diseases	A00-B99	402 (1.1) [1.0-1.2]	115 (1.1) [0.9-1.3]	208 (1.5) [1.3-1.7]	570 (1.7) [1.6-1.8]	1295 (1.4) [1.3-1.5]
Neoplasms	C00-D48	101 (0.3) [0.2-0.4]	54 (0.5) [0.4-0.6]	70 (0.5) [0.4-0.6]	239 (0.7) [0.6-0.8]	464 (0.5) [0.5-0.5]
Diseases of the blood and blood-forming organs and certain disorders involving the immune mechanism	D50-D89	40 (0.1) [0.1-0.1]	18 (0.2) [0.1-0.3]	24 (0.2) [0.1-0.3]	82 (0.2) [0.2-0.2]	164 (0.2) [0.2-0.2]
Endocrine, nutritional, and metabolic disorders	E00-E89	99 (0.3) [0.2-0.4]	68 (0.7) [0.5-0.9]	48 (0.4) [0.3-0.5]	211 (0.6) [0.5-0.7]	426 (0.5) [0.5-0.5]
Mental and behavioral disorders	F00-F99	466 (1.2) [1.1-1.3]	145 (1.4) [1.2-1.6]	371 (2.7) [2.4-3.0]	1234 (3.7) [3.5-3.9]	2216 (2.3) [2.2-2.4]
Diseases						
Nervous system	G00-G99	110 (0.3) [0.2-0.4]	47 (0.5) [0.4-0.6]	80 (0.6) [0.5-0.7]	309 (0.9) [0.8-1.0]	546 (0.6) [0.6-0.6]
Eye and adnexa	H00-H59	30 (0.1) [0.1-0.1]	11 (0.1) [0.0-0.2]	19 (0.1) [0.0-0.2]	72 (0.2) [0.2-0.2]	132 (0.1) [0.1-0.1]
Ear and mastoid process	H60-H95	30 (0.1) [0.1-0.1]	16 (0.2) [0.1-0.3]	28 (0.2) [0.1-0.3]	94 (0.3) [0.2-0.4]	168 (0.2) [0.2-0.2]
Circulatory system	I00-I99	101 (0.3) [0.2-0.4]	45 (0.4) [0.3-0.5]	66 (0.5) [0.4-0.6]	185 (0.6) [0.5-0.7]	397 (0.4) [0.4-0.4]
Respiratory system	J00-J99	641 (1.7) [1.6-1.8]	256 (2.5) [2.2-2.8]	410 (3.0) [2.7-3.3]	1257 (3.8) [3.6-4.0]	2564 (2.7) [2.6-2.8]
Digestive system	K00-K93	1185 (3.2) [3.0-3.4]	369 (3.6) [3.2-4.0]	796 (5.8) [5.4-6.2]	2075 (6.3) [6.0-6.6]	4425 (4.7) [4.6-4.8]
Skin and subcutaneous tissue system	L00-L099	315 (0.8) [0.7-0.9]	138 (1.3) [1.1-1.5]	168 (1.2) [1.0-1.4]	673 (2.0) [1.8-2.2]	1294 (1.4) [1.3-1.5]
Musculoskeletal system and connective tissue	M00-M99	289 (0.8) [0.7-0.9]	82 (0.8) [0.6-1.0]	161 (1.2) [1.0-1.4]	600 (1.8) [1.7-1.9]	1132 (1.2) [1.1-1.3]
Genitourinary system	N00-N99	416 (1.1) [1.0-1.2]	157 (1.5) [1.3-1.7]	264 (1.9) [1.7-2.1]	953 (2.9) [2.7-3.1]	1790 (1.9) [1.8-2.0]
Pregnancy, childbirth, and the puerperium	O00-O99	308 (0.8) [0.7-0.9]	153 (1.5) [1.3-1.7]	608 (4.5) [4.2-4.8]	2594 (7.9) [7.6-8.2]	3663 (3.9) [3.8-4.0]
Certain conditions originating in the perinatal period	P00-P96	NA^b^	NA^b^	NA^b^	NA^b^	NA^b^
Congenital malformations, deformations, and chromosomal abnormalities	Q00-Q99	NA^b^	NA^b^	NA^b^	NA^b^	NA^b^
Symptoms, signs, and abnormal clinical and laboratory findings, not elsewhere classified	R00-R99	639 (1.7) [1.6-1.8]	254 (2.5) [2.2-2.8]	446 (3.3) [3.0-3.6]	1547 (4.7) [4.5-4.9]	2886 (3.1) [3.0-3.2]
Injury, poisoning, and certain other consequences of external causes	S00-T98	1887 (5.0) [4.8-5.2]	634 (6.2) [5.7-6.7]	994 (7.3) [6.9-7.7]	2961 (9.0) [8.7-9.3]	6476 (6.9) [6.7-7.1]
Factors influencing health status and contact with health services	Z00-Z99	255 (0.7) [0.6-0.8]	89 (0.9) [0.7-1.1]	153 (1.1) [0.9-1.3]	535 (1.6) [1.5-1.7]	1032 (1.1) [1.0-1.2]
PPHs	NA	682 (1.8) [1.7-1.9]	296 (2.9) [2.6-3.2]	406 (3.0) [2.7-3.3]	1597 (4.8) [4.6-5.0]	2981 (3.2) [3.1-3.3]
CCCs	NA	241 (0.6) [0.5-0.7]	154 (1.5) [1.3-1.7]	200 (1.5) [1.3-1.7]	713 (2.2) [2.0-2.4]	1308 (1.4) [1.3-1.5]

Abbreviations: CCC, complex chronic condition; *ICD-10-AM*, *International Statistical Classification of Diseases and Related Health Problems, Tenth Revision, Australian Modification*; PPH; potentially preventable hospitalization.

^a^ Individuals may experience multiple admissions and therefore may fall into multiple *ICD-10-AM* chapters.

^b^ Data concealed due to cell size of fewer than 5 individuals.

### Sensitivity Analysis

Both sensitivity analyses were consistent with the main findings (eTable 5 and eTable 6 in the Supplement). Increasing observation periods of either parent or offspring WC increased the portion of the cohort who experienced IWC by approximately 3.5% (95% CI, 3.3%-3.7%). Additional sensitivity analysis was undertaken for individuals born in 1991 and 1995 to compare parental family structure defined by birth registration data to male and female parents recorded in the Centrelink relationships file. For 96.9% (95% CI, 96.7%-97.1%) of individuals who had a female parent recorded in the Centrelink relationships file, that female parent ID matched the mother on the birth registration. For 90.0% (95% CI, 89.7%-90.3%) of individuals with at least 1 male parent registered in the Centrelink relationships file, that male parent ID matched the coparent on the birth registration. Only 33.2% (95% CI, 32.7%-33.7%) of male coparents in birth registrations were recorded in the Centrelink relationships file as having a relationship to the individual.

## Discussion

This cohort study found that approximately 1 in 3 individuals (34.9%) born in South Australia from 1991 to 1995 experienced IWC, defined as means-tested payments designed to support more disadvantaged families raising children, and those offspring themselves. This is perhaps unsurprising, given cross-sectional survey estimates suggest 1 in 6 children in Australia younger than 15 years live in poverty.^3^ Our findings provide a nuanced understanding of IWC, as we considered welfare payments with eligibility requirements linked to socioeconomic disadvantage. If we took the same approach as previous research and considered any payment type, nearly half of all included individuals (48.9%) had experienced IWC. However, this estimate includes near universal payments that normalize the experience of IWC in society. While it is important that such universal supports exist, they have little meaningful interpretation for considering potential interventions^47^ to help break cycles of intergenerational disadvantage, which should focus on differences between individuals with parent-only WC and IWC.

We believe this is the first study to report the health burden of IWC using welfare payment data. Individuals with IWC had a 78% higher hospitalization rate compared with individuals with parent-only WC. We compared individuals with IWC with those experiencing parent-only WC, as they are the target group we would potentially intervene on to break the cycle of IWC.^47^ Of all individuals with IWC, 43% experienced at least 1 hospitalization between ages 11 and 20 years, most commonly related to injury, respiratory conditions, digestive conditions, mental health, and pregnancy. While welfare receipt has been associated with poorer mental health outcomes,^11,29,31,34^ our study showed individuals with IWC experienced even higher mental health hospitalization rates compared with individuals with WC experienced only in 1 generation. We expected individuals with IWC to have higher injury hospitalization rates, given injury hospitalization is more frequent in individuals from disadvantaged backgrounds^45,46^ but this had not been quantified. Individuals with IWC having higher hospitalization rates related to pregnancy and childbirth reflects the inclusion of welfare payments associated with parenting.^21^ A higher proportion of individuals with IWC experienced PPHs, which are also associated with socioeconomic disadvantage.^45^ While individuals with IWC were more likely to experience a hospitalization relating to a CCC, the overall proportion was small (age 11-15 years: 1.9%; age 16-20 years: 2.2%). This suggests that most individuals with IWC were likely to be receiving welfare payments owing to low income or socioeconomic disadvantage rather than chronic poor health.

Offspring of a parent with WC were 2.4-fold more likely to experience their own WC compared with individuals with no parental WC, which is consistent with previous findings.^21,23,24,25^ Progress in breaking cycles of entrenched disadvantage is slow.^13,20^ We found that one-third of the population in South Australia experienced IWC and that individuals with IWC contributed a higher hospital burden compared with all other WC types. This suggests that breaking the intergenerational cycle of WC is important for reducing hospital burden. Our analysis highlights the health burden of IWC for the first time, to our knowledge. Current policy discussions of breaking the cycle^13,19,20^ of IWC have occurred within agencies responsible for the welfare system. These agencies bear the direct cost of administering welfare, but it is not the only place where IWC burden is experienced. Our findings reveal that the health system bears some of the IWC burden but has not previously been engaged in policy discussions of breaking the cycle of IWC. Our analysis frames the policy problem of IWC in a new light by highlighting the downstream cost shift onto the health system.

### Aboriginal and Torres Strait Islander People and Their Data

Aboriginal and Torres Strait Islander individuals and families are over-represented in the welfare system.^9^ Typically, outcomes for Aboriginal and Torres Strait Islander individuals and families are compared with outcomes of non-Indigenous individuals and families. This is done with little consideration of the context of the phenomena of interest. The Australian welfare system was constructed to improve well-being, but the conceptual model it was built on was not created for Aboriginal and Torres Strait Islander people. If we were to take an Aboriginal and Torres Strait Islander world view, we would need to use a different conceptual model of what “welfare” means. Such a model would consider family and kinship structure, the sharing of resources, and understand that money does not necessarily have the same meaning, nor reflect the holistic concept of well-being, for Aboriginal and Torres Strait Islander families and communities.^48^ A collective decision was made after consultation among the author group, one of whom is an Aboriginal and Torres Strait Islander researcher, to apply the principles of Indigenous Data Sovereignty to control the Aboriginal and Torres Strait Islander data in the BEBOLD platform. The South Australian Aboriginal community were not involved in informing this research and so their data were not included. Globally, Indigenous populations are enacting Indigenous Data Sovereignty, defined as “the rights of Indigenous Peoples to determine the means of collection, access, analysis, interpretation, management, dissemination and re-use of data pertaining to the Indigenous Peoples from whom it has been derived, or to whom it relates.”^37^ These principles are fundamental and should be considered and applied by all researchers.

### Limitations

This study has some limitations. We defined WC as a parent or offspring having received an eligible payment. Consideration of the amount and duration of payment are topics for future research. However, it is unlikely that individuals only received payments for a short period of time, because payments included under the means-tested construct are intended for longer-term income support. A study by Cobb-Clark and colleagues^21^ found the likelihood of offspring experiencing IWC was more closely related to whether the parent received a welfare payment at all, rather than the amount, type, intensity, or duration.

Our study defined family structure at the time of childbirth using South Australia birth registrations, which contain records of mother and coparent (if acknowledged). Centrelink records contain additional information regarding relationship that can imply family structure. We found a high proportion of mothers (97%) and coparents (90%) from birth registration matched the Centrelink relationships file where recorded. Only 33% of birth registration coparents were recorded in the Centrelink relationships file. If we were to define family structure using the Centrelink relationships file, it would not be possible to know which parent relationship reflected the primary caregiver, and our view of 2-parent families would be substantially limited. We have some confidence in the veracity of using birth registration data given that most families (73%) with children aged 0 to 17 years in 2006 to 2007, both coupled and 1-parent, were intact,^49^ meaning that they had not changed since the time of birth.

Our estimates of WC type and IWC were limited by the data available. Sensitivity analysis showed that by increasing observation periods of either parent or offspring WC, the proportion of the cohort who experienced IWC increased by approximately 3.5%. While there is no reason to suspect that the association between WC and hospitalizations would be different if we could examine the entire life course of offspring, our estimates of IWC should be considered an underestimation.

Private hospital data are not available for linkage in South Australia, but public hospitals account for 92% of admissions classified as an emergency and the only children’s hospital in South Australia is a public hospital,^50^ suggesting that we have captured most hospitalizations in our analysis. The exclusion of private hospital data may mean we underestimated cumulative incidence, but we would not expect the associations between WC and hospital burden to differ between individuals attending public and private hospitals.

There is possible loss to follow-up in hospitalization outcomes due to individuals moving interstate. Hospitalization rates should therefore be considered an underestimate. We do not expect this underestimate to vary across the different WC groups.

## Conclusions

This cohort study examined the health burden of IWC using a definition of means-tested payments designed to support disadvantaged families and their children. Individuals with IWC had a 78% higher hospitalization rate compared with individuals with parent-only WC, and 190% higher than those with no WC. The hospital burden associated with individuals with IWC is large, with 43% of individuals with IWC experiencing at least 1 hospitalization between ages 11 and 20 years. Individuals with means-tested IWC comprised one-third of the study population, but accounted for more than half of all hospital admissions from ages 11 to 20 years. Common causes of hospitalizations included PPHs, injuries, mental health, pregnancy, and childbirth.

## Notes

Supplement. **eFigure 1.** Map of Parent and Offspring Welfare Contact by Birth Cohort, Calendar Year, and Offspring Age **eFigure 2.** Study Population Flowchart **eTable 1.** Indicators of Sociodemographic Disadvantage at Birth by Welfare Contact (WC) for Offspring Born From 1991 to 1995 **eTable 2.** Welfare Payment Types Included Under the Means-Tested Definition **eTable 3.** Cause Specific Admission Rates per 1000 Person-Years (PY) for Offspring Born From 1991 to 1995, Aged 11 to 15 Years by Welfare Contact (WC) **eTable 4.** Cause Specific Admission Rates per 1000 Person-Years (PY) for Offspring Born From 1991 to 1995, Aged 16 to 20 Years by Welfare Contact (WC) **eTable 5.** Means-Tested Welfare Contact (WC) Groups When Offspring WC is Measured at Ages 16 to 20 Years or 16 to 24 Years For Offspring Born in 1991 **eTable 6.** Means-Tested Welfare Contact (WC) Groups When Parent WC is Measured at Offspring Age 11-15 Years and 7-15 Years For Offspring Born in 1995 **eTable 7.** Person-Years (PY) Used to Calculate All-Cause Hospital Admission Rates per 1000 PY for Offspring Born From 1991 to 1995 by Welfare Contact (WC)
