# Supplementary material for: The Hospital Burden Associated With Intergenerational Contact With the Welfare System in Australia
Source: JAMA Netw Open. 2022 Aug 5;5(8):e2226203. doi: 10.1001/jamanetworkopen.2022.26203 (PMC9356314; doi:10.1001/jamanetworkopen.2022.26203)
Supplement: Supplement. — eFigure 1. Map of Parent and Offspring Welfare Contact by Birth Cohort, Calendar Year, and Offspring Age eFigure 2. Study Population Flowchart eTable 1. Indicators of Sociodemographic Disadvantage at Birth by Welfare Contact (WC) for Offspring Born From 1991 to 1995 eTable 2. Welfare Payment Types Included Under the Means-Tested Definition eTable 3. Cause Specific Admission Rates per 1000 Person-Years (PY) for Offspring Born From 1991 to 1995, Aged 11 to 15 Years by Welfare Contact (WC) eTable 4. Cause Specific Admission Rates per 1000 Person-Years (PY) for Offspring Born From 1991 to 1995, Aged 16 to 20 Years by Welfare Contact (WC) eTable 5. Means-Tested Welfare Contact (WC) Groups When Offspring WC is Measured at Ages 16 to 20 Years or 16 to 24 Years For Offspring Born in 1991 eTable 6. Means-Tested Welfare Contact (WC) Groups When Parent WC is Measured at Offspring Age 11-15 Years and 7-15 Years For Offspring Born in 1995 eTable 7. Person-Years (PY) Used to Calculate All-Cause Hospital Admission Rates per 1000 PY for Offspring Born From 1991 to 1995 by Welfare Contact (WC) [file jamanetwopen-e2226203-s001.pdf]

Supplemental Online Content

Procter AM, Chittleborough CR, Pilkington RM, Pearson O, Montgomerie A, Lynch JW. The hospital burden associated with intergenerational contact with the welfare system in Australia. *JAMA Netw Open*. 2022;5(8):e2226203.
doi:10.1001/jamanetworkopen.2022.26203

eFigure 1. Map of Parent and Offspring Welfare Contact by Birth Cohort, Calendar Year, and Offspring Age

eFigure 2. Study Population Flowchart

eTable 1. Indicators of Sociodemographic Disadvantage at Birth by Welfare Contact (WC) for Offspring Born From 1991 to 1995

eTable 2. Welfare Payment Types Included Under the Means-Tested Definition

eTable 3. Cause Specific Admission Rates per 1000 Person-Years (PY) for Offspring Born From 1991 to 1995, Aged 11 to 15 Years by Welfare Contact (WC)

eTable 4. Cause Specific Admission Rates per 1000 Person-Years (PY) for Offspring Born From 1991 to 1995, Aged 16 to 20 Years by Welfare Contact (WC)

eTable 5. Means-Tested Welfare Contact (WC) Groups When Offspring WC is Measured at Ages 16 to 20 Years or 16 to 24 Years For Offspring Born in 1991

eTable 6. Means-Tested Welfare Contact (WC) Groups When Parent WC is Measured at Offspring Age 11-15 Years and 7-15 Years For Offspring Born in 1995

eTable 7. Person-Years (PY) Used to Calculate All-Cause Hospital Admission Rates per 1000 PY for Offspring Born From 1991 to 1995 by Welfare Contact (WC)

This supplemental material has been provided by the authors to give readers additional information about their work.

© 2022 Procter AM et al. *JAMA Network Open.*

eFigure 1. Map of Parent and Offspring Welfare Contact by Birth Cohort, Calendar Year, and Offspring Age

Parent welfare contact- observed at offspring age 11-15 years

Offspring welfare contact- observed at offspring age 16-20 years

		Calendar year																									
		1991	1992	1993	1994	1995	1996	1997	1998	1999	2000	2001	2002	2003	2004	2005	2006	2007	2008	2009	2010	2011	2012	2013	2014	2015	2016
Birth cohort	1991	0	1	2	3	4	5	6	7	8	9	10	11	12	13	14	15	16	17	18	19	20	21	22	23	24	25
	1992		0	1	2	3	4	5	6	7	8	9	10	11	12	13	14	15	16	17	18	19	20	21	22	23	24
	1993			0	1	2	3	4	5	6	7	8	9	10	11	12	13	14	15	16	17	18	19	20	21	22	23
	1994				0	1	2	3	4	5	6	7	8	9	10	11	12	13	14	15	16	17	18	19	20	21	22
	1995					0	1	2	3	4	5	6	7	8	9	10	11	12	13	14	15	16	17	18	19	20	21
	1996						0	1	2	3	4	5	6	7	8	9	10	11	12	13	14	15	16	17	18	19	20
	1997							0	1	2	3	4	5	6	7	8	9	10	11	12	13	14	15	16	17	18	19
	1998								0	1	2	3	4	5	6	7	8	9	10	11	12	13	14	15	16	17	18
	1999									0	1	2	3	4	5	6	7	8	9	10	11	12	13	14	15	16	17
	2000										0	1	2	3	4	5	6	7	8	9	10	11	12	13	14	15	16
	2001											0	1	2	3	4	5	6	7	8	9	10	11	12	13	14	15
	2002												0	1	2	3	4	5	6	7	8	9	10	11	12	13	14

Parent welfare contact

Offspring welfare contact

© 2022 Procter AM et al. *JAMA Network Open.*

eFigure 2. Study Population Flowchart

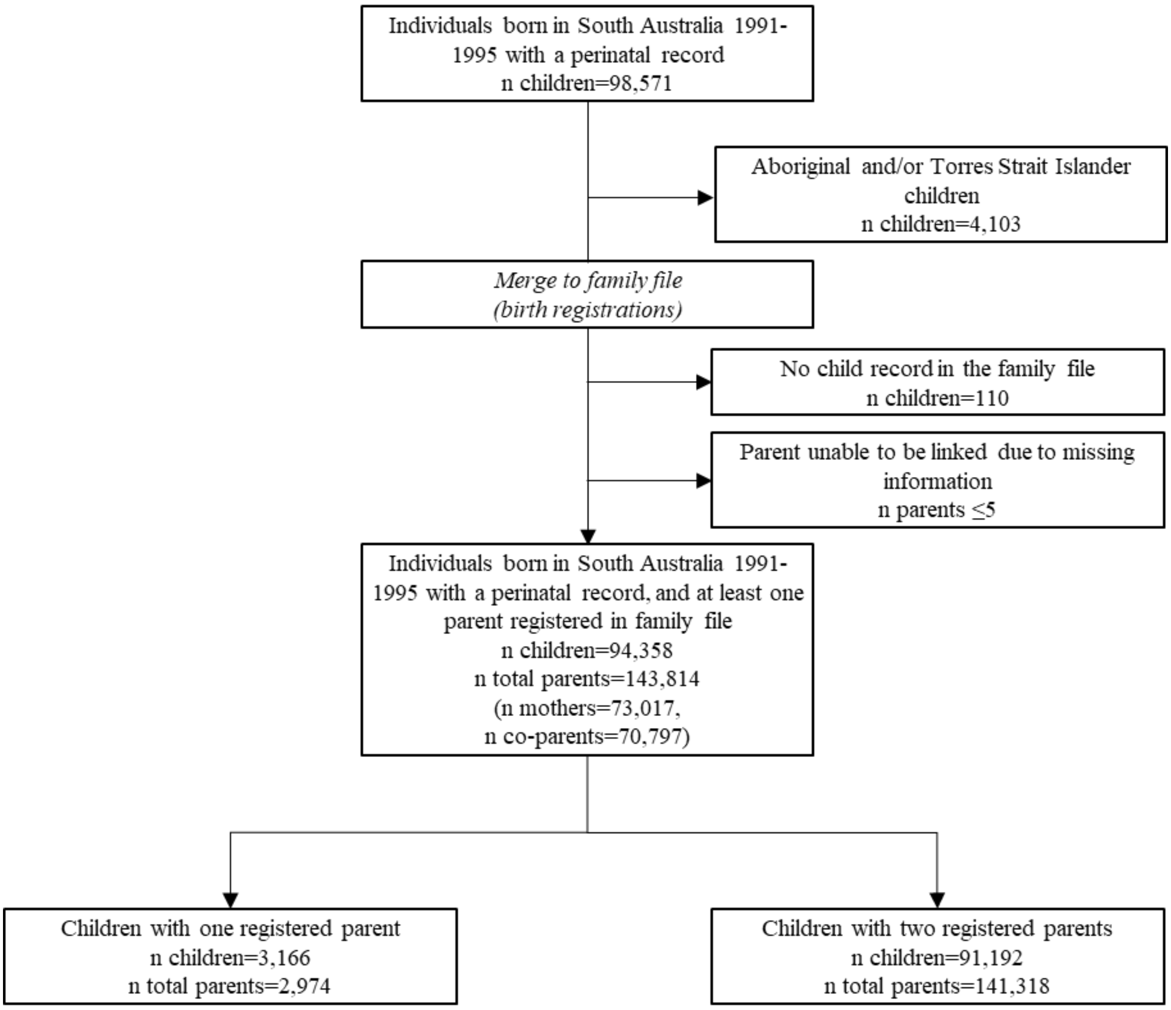

© 2022 Procter AM et al. *JAMA Network Open.*

eTable 1. Indicators of Sociodemographic Disadvantage at Birth by Welfare Contact (WC) for Offspring Born From 1991 to 1995

Indicators of sociodemographic and health disadvantage	No WC n= 37,507	Parent only WC n= 10,260	Offspring only WC n= 13,622	Intergenerational WC n= 32,969	Overall n= 94,358
	%	%	%	%	%
**Maternal age (years) at birth of child**					
<20	1.2	5.4	3.1	9.4	4.8
20-24	11.9	23.1	17.1	26.7	19.0
25-29	37.7	37.3	37.3	32.0	35.6
30-34	36.3	25.3	30.6	21.9	29.2
35-39	11.4	7.7	10.1	8.5	9.8
40+	1.5	1.2	1.8	1.5	1.5
**Maternal age (years) at first birth**					
<20	1.5	6.9	3.7	11.6	5.9
20-24	14.4	25.0	19.6	28.0	21.0
25-29	39.8	37.7	38.6	31.4	36.5
30-34	33.1	22.7	27.7	20.2	26.7
35-39	9.8	6.7	8.8	7.6	8.6
40+	1.3	1.1	1.5	1.3	1.3
**Mother's partner status**					
No-partner	5.4	13.4	9.1	22.6	12.8
Partner	#	#	#	77.3	87.2
**Jobless family**					
Family in labour force	95.4	87.1	91.4	72.2	85.8
Family not in labour force	4.3	12.4	8.2	27.0	13.7
**Child sex**					
Male	52.7	55.8	46.2	51.0	51.5
Female	47.3	44.2	53.8	49.0	48.5
**IRSAD[a] quintile**					
1 Most advantaged	25.4	17.3	14.0	10.2	17.6
2	23.6	20.8	18.5	16.1	19.9
3	15.1	15.7	16.3	15.5	15.5
4	16.4	18.0	23.3	19.7	18.7
5 Most disadvantaged	18.9	27.9	27.0	38.0	27.7
**Mother born in Australia**					
No	19.3	17.0	18.7	20.1	19.3

© 2022 Procter AM et al. *JAMA Network Open.*

Yes	80.6	82.9	81.2	79.8	80.7
**Preterm birth**					
No (Gestational age 37+ weeks)	#	#	#	#	93.4
Yes (Gestational age <37 weeks)	5.9	7.7	5.8	7.4	6.6
**Low birthweight**					
No (weight 2500+ grams)	#	#	94.6	92.9	#
Yes (weight <2500 grams)	5.0	6.5	5.4	7.1	6.0
Total	100.0	100.0	100.0	100.0	100.0

[a] Index of Relative Socio-economic Advantage and Disadvantage.
Note: some % do not sum to 100% due to <1% of missing data on some perinatal characteristic response categories.
# represents cells redacted to prevent back calculation of cell size n<5

Characteristics examined in the analysis included: maternal age at birth, maternal age at mother's first birth, mother's partner status, mother born in Australia, child sex, low birth weight (<2,500 grams), and preterm birth (gestational age <37 weeks). Families not in the labour force included two parent families, or mothers with no partner, where no parent was recorded as being in the labour force. Using postcode of mother's usual residence, area-level socioeconomic disadvantage was determined using the Index of Relative Socioeconomic Advantage and Disadvantage (IRSAD).[1]

[1]*Australian Bureau of Statistics. 2033.0.55.001 - Census of Population and Housing: Socio-Economic Indexes for Areas (SEIFA), Australia, 2011. Canberra: Commonwealth of Australia; [cited 2019 11 January 2019]. Available from: http://www.abs.gov.au/ausstats/abs@.nsf/Lookup/2033.0.55.001main+features100132011*

© 2022 Procter AM et al. *JAMA Network Open.*

eTable 2. Welfare Payment Types Included Under the Means-Tested Definition

**Payment type**	**Intention**	**Means testing**	**Activity tested/ mutual obligations**
Carer Allowance	Income supplement paid to an individual who provides daily care and attention at home to a person with disability/illness.	Yes	No
Carer Payment	Paid to an individual who is providing constant care for person(s) with disability/illness.	Yes	No
Disability Support Pension (DSP)	Individual diagnosed with permanent physical, intellectual, or psychiatric impairment that meets specific manifest eligibility criteria (e.g. permanently blind)	Yes	No
	Individual diagnosed with permanent physical, intellectual, or psychiatric impairment which meets minimum impairment threshold AND cannot work for ≥15h/week for 2 years.		Potentially-dependent on level of impairment due to disability.
Parenting payment partnered	Principal carer of ≥1 child who is ≤6 years old	Yes	Yes
Parenting payment single	Principal carer of ≥1 child who is ≤8 years old	Yes	Yes
Newstart Allowance	Unemployed, looking for work, and willing to work	Yes	Yes
Newstart Mature Age Allowance	Unemployed, looking for work, and willing to work, aged 55 years and over	Yes	Yes
Youth Allowance	Looking for employment or doing approved activities (e.g. full-time student or Australian apprentice)	Yes	Yes
Partner allowance	Member of a couple where partner received qualifying pension (e.g. Newstart, DSP, carer allowance etc.) and recipient has no recent workforce experience.	Yes	Yes
Wife Pension DSP (wife of a disability support pension)	Income support payment for female partners of people receiving Disability support pension.	Yes	No

© 2022 Procter AM et al. *JAMA Network Open.*

eTable 3. Cause Specific Admission Rates per 1000 Person-Years (PY) for Offspring Born From 1991 to 1995, Aged 11 to 15 Years by Welfare Contact (WC)

Condition type (respective ICD-10-AM codes)	No WC py=187,535		Parent only WC py=51,300		Offspring only WC py=68,110		Intergenerational WC py=164,845		Overall py=471,790	
	n	Rate per 1,000 py	n	Rate per 1,000 py	n	Rate per 1,000 py	n	Rate per 1,000 py	n	Rate per 1,000 py
Certain infectious & parasitic diseases (A00-B99)	307	1.6	154	3.0	135	2.0	454	2.8	1,050	2.2
Neoplasms (C00-D48)	101	0.5	322	6.3	73	1.1	499	3.0	995	2.1
Diseases of the blood and blood-forming organs and certain disorders involving the immune mechanism (D50-D89)	41	0.2	212	4.1	12	0.2	229	1.4	494	1.0
Endocrine, nutritional and metabolic disorders (E00-E89)	159	0.8	428	8.3	71	1.0	643	3.9	1,301	2.8
Mental and behavioural disorders (F00-F99)	207	1.1	63	1.2	127	1.9	702	4.3	1,099	2.3
Diseases of the nervous system (G00-G99)	126	0.7	73	1.4	67	1.0	442	2.7	708	1.5
Diseases of the eye and adnexa (H00-H59)	39	0.2	8	0.2	22	0.3	114	0.7	183	0.4
Disease of the ear and mastoid process (H60-H95)	87	0.5	48	0.9	66	1.0	285	1.7	486	1.0
Diseases of the circulatory system (I00-I99)	86	0.5	42	0.8	39	0.6	137	0.8	304	0.6
Diseases of the respiratory system (J00-J99)	610	3.3	296	5.8	395	5.8	1,636	9.9	2,937	6.2
Diseases of the digestive system (K00-K93)	854	4.6	345	6.7	450	6.6	1,430	8.7	3,079	6.5
Diseases of the skin and subcutaneous tissue system (L00-L099)	289	1.5	96	1.9	166	2.4	625	3.8	1,176	2.5
Diseases of the musculoskeletal system and connective tissue (M00-M99)	236	1.3	110	2.1	116	1.7	493	3.0	955	2.0
Diseases of the genitourinary system (N00-N99)	231	1.2	106	2.1	110	1.6	412	2.5	859	1.8
Pregnancy, childbirth and the puerperium (O00-O99)	7	0.0	6	0.1	29	0.4	182	1.1	224	0.5
Certain conditions originating in the perinatal period (P00-P96)	0	0.0	0	0.0	0	0.0	0	0.0	0	0.0
Congenital malformations, deformations and chromosomal abnormalities (Q00-Q99)	96	0.5	50	1.0	45	0.7	266	1.6	457	1.0
Symptoms, signs and abnormal clinical and laboratory findings, not elsewhere classified (R00-R99)	518	2.8	200	3.9	258	3.8	1,054	6.4	2,030	4.3
Injury, poisoning and certain other consequences of external causes (S00-T98)	1,977	10.5	672	13.1	912	13.4	2,787	16.9	6,348	13.5

© 2022 Procter AM et al. *JAMA Network Open.*

Factors influencing health status and contact with health services (Z00-Z99)	430	2.3	272	5.3	196	2.9	536	3.3	1,434	3.0
Potentially preventable hospitalisations (PPHs)	832	4.4	341	6.6	409	6.0	1,790	10.9	3,372	7.1
Complex Chronic Conditions (CCCs)	248	1.3	1,039	20.3	119	1.7	1,349	8.2	2,754	5.8
Total	6,401	34.1	3,503	68.3	3,289	48.3	12,926	78.4	26,119	55.4

© 2022 Procter AM et al. *JAMA Network Open.*

eTable 4. Cause Specific Admission Rates per 1000 Person-Years (PY) for Offspring Born From 1991 to 1995, Aged 16 to 20 Years by Welfare Contact (WC)

Condition type (respective ICD-10-AM codes)	No WC py=187,535		Parent only WC py=51,300		Offspring only WC py=68,110		Intergenerational WC py=164,845		Overall py=471,790	
	n	Rate per 1,000 py	n	Rate per 1,000 py	n	Rate per 1,000 py	n	Rate per 1,000 py	n	Rate per 1,000 py
Certain infectious & parasitic diseases (A00-B99)	440	2.3	124	2.4	222	3.3	654	4.0	1,440	3.1
Neoplasms (C00-D48)	224	1.2	149	2.9	150	2.2	525	3.2	1,048	2.2
Diseases of the blood and blood-forming organs and certain disorders involving the immune mechanism (D50-D89)	77	0.4	134	2.6	46	0.7	398	2.4	655	1.4
Endocrine, nutritional and metabolic disorders (E00-E89)	160	0.9	139	2.7	99	1.5	700	4.2	1,098	2.3
Mental and behavioural disorders (F00-F99)	665	3.5	188	3.7	817	12.0	2,372	14.4	4,042	8.6
Diseases of the nervous system (G00-G99)	145	0.8	68	1.3	134	2.0	534	3.2	881	1.9
Diseases of the eye and adnexa (H00-H59)	35	0.2	12	0.2	35	0.5	#	#	171	0.4
Disease of the ear and mastoid process (H60-H95)	33	0.2	19	0.4	37	0.5	135	0.8	224	0.5
Diseases of the circulatory system (I00-I99)	124	0.7	75	1.5	90	1.3	242	1.5	531	1.1
Diseases of the respiratory system (J00-J99)	769	4.1	309	6.0	498	7.3	1,726	10.5	3,302	7.0
Diseases of the digestive system (K00-K93)	1,374	7.3	481	9.4	956	14.0	2,536	15.4	5,347	11.3
Diseases of the skin and subcutaneous tissue system (L00-L099)	513	2.7	181	3.5	245	3.6	936	5.7	1,875	4.0
Diseases of the musculoskeletal system and connective tissue (M00-M99)	359	1.9	112	2.2	219	3.2	755	4.6	1,445	3.1
Diseases of the genitourinary system (N00-N99)	495	2.6	188	3.7	363	5.3	1,203	7.3	2,249	4.8
Pregnancy, childbirth and the puerperium (O00-O99)	393	2.1	215	4.2	1,009	14.8	4,918	29.8	6,535	13.9
Certain conditions originating in the perinatal period (P00-P96)	#	#	#	#	#	#	#	#	#	#
Congenital malformations, deformations and chromosomal abnormalities (Q00-Q99)	#	#	#	#	#	#	#	#	#	#
Symptoms, signs and abnormal clinical and laboratory findings, not elsewhere classified (R00-R99)	727	3.9	301	5.9	568	8.3	2,015	12.2	3,611	7.7
Injury, poisoning and certain other consequences of external causes (S00-T98)	2,362	12.6	789	15.4	1,333	19.6	4,068	24.7	8,552	18.1

© 2022 Procter AM et al. *JAMA Network Open.*

Factors influencing health status and contact with health services (Z00-Z99)	493	2.6	292	5.7	444	6.5	1,697	10.3	2,926	6.2
Potentially preventable hospitalisations (PPHs)	754	4.0	361	7.0	471	6.9	2,041	12.4	3,627	7.7
Complex Chronic Conditions (CCCs)	4,171	8.8	584	3.1	579	11.3	645	9.5	2,366	14.4
Total	9,458	50.4	3,808	74.2	7,290	107.0	25,687	155.8	46,243	98.0

# represents cells concealed due to cell size n<5

© 2022 Procter AM et al. *JAMA Network Open.*

eTable 5. Means-Tested Welfare Contact (WC) Groups When Offspring WC is Measured at Ages 16 to 20 Years or 16 to 24 Years For Offspring Born in 1991

WC when child WC is measured at ages 16-20 years	WC when offspring WC is measured at ages 16-24 years													
	No WC			Parent only WC			Offspring only WC			IWC			Total	
	n	Row%	Col%	n	Row%	Col%	n	Row%	Col%	n	Row%	Col%	n	Col%
No WC	5,266	70.5	100.0	-	-	-	2,204	29.5	46.7	-	-	-	7,470	39.9
Parent only WC	-	-	-	1,554	69.7	100.0	-	-	-	677	30.3	9.4	2,231	11.9
Offspring only WC	-	-	-	-	-	-	2,517	100.0	53.3	-	-	-	2,517	13.4
IWC	-	-	-	-	-	-	-	-	-	6,526	100.0	90.6	6,526	34.8
Total	5,266	28.1	100.0	1,554	8.3	100.0	4,721	25.2	100.0	7,203	38.4	100.0	18,744	100.0

eTable 5 compares offspring WC measured at 16-20 years to an extended view measured at 16-24 years for individuals born in 1991. Under the extended view, an additional n=2,204 individuals (11.8% of the birth cohort) experienced offspring only WC and an additional n=677 individuals (3.6% of birth cohort) experienced IWC.

© 2022 Procter AM et al. *JAMA Network Open.*

eTable 6. Means-Tested Welfare Contact (WC) Groups When Parent WC is Measured at Offspring Age 11-15 Years and 7-15 Years For Offspring Born in 1995

WC when parent WC is measured at ages 11-15 years	WC when parent WC is measured at ages 7-15 years													
	No WC			Parent only WC			Offspring only WC			IWC			Total	
	n	Row%	Col%	n	Row%	Col%	n	Row%	Col%	n	Row%	Col%	n	Col%
No WC	6,697	88.0	100.0	912	12.0	32.3	-	-	-	-	-	-	7,609	40.8
Parent only WC	-	-	-	1,913	100.0	67.7	-	-	-	-	-	-	1,913	10.3
Offspring only WC	-	-	-	-	-	-	2,080	75.9	100.0	659	24.1	9.4	2,739	14.7
IWC	-	-	-	-	-	-	-	-	-	6,386	100.0	90.6	6,386	34.2
Total	6,697	35.9	100.0	2,825	15.1	100.0	2,080	11.2	100.0	7,045	37.8	100.0	18,647	100.0

eTable 6 compares parent WC measured for individuals born in 1995 when the individual was 11-15 years to an extended view measured when the individual was 7-15 years. An additional n=912 individuals (4.9% of the birth cohort) experienced parent only WC and an additional n=659 individuals (3.5% of the birth cohort) experienced IWC. Increasing observation periods of either parent or offspring WC increased the portion of the cohort who experiences IWC by ~3.5%.

© 2022 Procter AM et al. *JAMA Network Open.*

eTable 7. Person-Years (PY) Used to Calculate All-Cause Hospital Admission Rates per 1000 PY for Offspring Born From 1991 to 1995 by Welfare Contact (WC)

		No WC			Parent only WC			Offspring only WC			Intergenerational WC			Overall		
Birth cohorts	Age years	py	n	rate per 1,000 py	py	n	rate per 1,000 py	py	n	rate per 1,000 py	py	n	rate per 1,000 py	py	n	rate per 1,000 py
1991-1995	11	37,507	1,066	28.4	10,260	692	67.4	13,622	510	37.4	32,969	2,161	65.5	94,358	4,429	46.9
1991-1995	12	37,507	1,187	31.6	10,260	615	59.9	13,622	613	45.0	32,969	2,184	66.2	94,358	4,599	48.7
1991-1995	13	37,507	1,275	34.0	10,260	664	64.7	13,622	624	45.8	32,969	2,335	70.8	94,358	4,898	51.9
1991-1995	14	37,507	1,377	36.7	10,260	766	74.7	13,622	687	50.4	32,969	2,845	86.3	94,358	5,675	60.1
1991-1995	15	37,507	1,496	39.9	10,260	766	74.7	13,622	855	62.8	32,969	3,401	103.2	94,358	6,518	69.1
1991-1995	16	37,507	1,669	44.5	10,260	732	71.3	13,622	1,275	93.6	32,969	4,275	129.7	94,358	7,951	84.3
1991-1995	17	37,507	1,892	50.4	10,260	874	85.2	13,622	1,339	98.3	32,969	4,842	146.9	94,358	8,947	94.8
1991-1995	18	37,507	1,969	52.5	10,260	707	68.9	13,622	1,463	107.4	32,969	5,242	159.0	94,358	9,381	99.4
1991-1995	19	37,507	1,954	52.1	10,260	724	70.6	13,622	1,546	113.5	32,969	5,637	171.0	94,358	9,861	104.5
1991-1995	20	37,507	1,974	52.6	10,260	771	75.1	13,622	1,667	122.4	32,969	5,691	172.6	94,358	10,103	107.1
1991-1995	21	37,507	2,130	56.8	10,260	851	82.9	13,622	1,747	128.2	32,969	5,568	168.9	94,358	10,296	109.1
1991-1994	22	29,898	1,650	55.2	8,347	730	87.5	10,883	1,268	116.5	26,583	4,917	185.0	75,711	8,565	113.1
1991-1993	23	22,501	1,372	61.0	6,502	643	98.9	8,048	952	118.3	19,826	3,903	196.9	56,877	6,870	120.8
1991-1992	24	15,024	916	61.0	4,424	360	81.4	5,190	621	119.7	13,227	2,774	209.7	37,865	4,671	123.4
1991	25	7,470	523	70.0	2,231	189	84.7	2,517	286	113.6	6,526	1,488	228.0	18,744	2,486	132.6
Total	11-25	487,470	22,450	46.1	134,364	10,084	75.0	176,480	15,453	87.6	428,821	57,263	133.5	1,227,135	105,250	85.8

© 2022 Procter AM et al. *JAMA Network Open.*
